# Rapid synergistic cloud point extraction of copper in environmental samples with greenness and toxicity evaluation using a triazole based Schiff base

**DOI:** 10.1038/s41598-026-35659-3

**Published:** 2026-02-03

**Authors:** Magda A. Akl, Eslam A. Ghaith, Aya G. Mostafa

**Affiliations:** Department of Chemistry, Faculty of Science, Mansoura University, Mansoura, 31556 Egypt

**Keywords:** Schiff base, TX-114, Cloud point extraction, Greenness, BAGI, RS-CPE, ICP-OES, Copper, Chemistry, Environmental sciences

## Abstract

Herein, a novel copper-triazole derivative Schiff base nanocomplex (HIT-Cu^2+^) was obtained via the reaction of the 3-(2-(4-amino-5-mercapto-4 H-1,2,4-triazol-3-yl)hydrazineylidene)indolin-2-one (HIT) Schiff base with CuCl_2_.6H_2_O. The non-ionic surfactant Triton X-114 (TX-114) was used as the extractant. The HIT, HIT-Cu^2+^, and HIT-Cu^2+^ in the organic layer were characterized by several techniques, including CHNS elemental analysis, ultraviolet-visible spectroscopy (UV–Vis), Fourier transform infrared spectroscopy (FT-IR), and transmission electron microscopy (TEM). The TEM results revealed the formation of the HIT-Cu^2+^ nanocomplex, with a particle size ranging from 13 to 25 nm. Moreover, an improved preconcentration method, named rapidly synergistic cloud point extraction (RS-CPE), was established for Cu^2+^ preconcentration and determination to overcome the issues investigated in CPE. Decanol acted as a cloud point revulsant and synergistic reagent, lowering the cloud point temperature (CPT) of TX-114, so that CPE could be done at room temperature without heating. Compared with traditional CPE (about 30 min for heating, incubation and cooling), RS-CPE was accomplished in 1 min with considerably high extraction efficiency. The improved extraction technique, RS-CPE, was combined with ICP-OES to improve the analytical performance and expand the application of ICP-OES determination. Various parameters that influence the Cu^2+^ removal efficiency (R, %) utilizing HIT Schiff base were investigated, including the solution’s pH and volume, the concentration of HIT and Cu^2+^, type of surfactant, TX-114 concentration, equilibrium temperature and time, interfering ions, and centrifugation time and rate. At optimum conditions, the Cu^2+^ linear range, the correlation coefficient, the limit of detection (LOD), and the limit of quantification (LOQ) were 0.5–5 µg.mL^− 1^, 0.9999, 0.018, and 0.054 µg/L, respectively. The investigated RS-CPE/ICP-OES approach was applied to real water and pharmaceutical samples, achieving recoveries higher than 97% with a preconcentration factor of 125. The HIT-Cu^2+^ CPE mechanism in the organic layer of TX-114 is elucidated. The greenness of the methods was evaluated using two recent methods: the Analytical Greenness (AGREE) calculator and the Blue Applicability Green Index (BAGI), which produced results in the ranges of 0.64–0.71 and 60-67.5, respectively. These results are strongly aligned with both green ideals and environmentally friendly practices. The predicted toxicity of HIT and HIT-Cu^2+^ nanocomplex was evaluated using PROTOX 3 software.

**Supplementary Information:**

The online version contains supplementary material available at 10.1038/s41598-026-35659-3.

## Introduction

Copper, which occupies positions 29 and 26 in the periodic table and in the lithosphere, respectively, is a necessary trace element for almost all living organisms, as it is a key constituent of several enzymes, such as Complex IV and L-ascorbate oxidase^1,2^. Naturally, copper occurs in minerals (~ 240) like cuprite, chalcocite, azurite, and malachite^3^. As copper has valuable properties and several applications, it is regarded as one of the most widely utilized metals^4,5^. Copper exists in three forms: copper metal, cuprous ion, and cupric ion, with oxidation states of 0, 1+, and 2+, respectively. Cu^2+^ is widely utilized in several industries, including the steel industry, painting, mining, and electroplating. Cu^2+^ has been identified as the most hazardous and prevalent element in the environment. The deficiency or extreme increase of copper in the body may lead to health risks, as its deficiency may cause anemia. In contrast, the extreme increase can result in acute gastrointestinal symptoms, liver enzymes inactivation, and even movement problems in some patients with copper overdose^6^. Environmental concentrations of Cu^2+^ as low as 0.01 mg L^− 1^ for invertebrates and 0.15 mg L^− 1^ for fish are considered toxic. Therefore, precise quantification of trace copper levels in environmental samples is essential^7,8^. Regulatory agencies have established strict guidelines for copper intake. The World Health Organisation (WHO) and the United States Environmental Protection Agency (US-EPA) recommend a maximum of 2 mg per day in drinking water. Additionally, the Food and Nutrition Board has set an upper limit of 10 mg per day for total copper consumption^9^. The European Food Safety Authority (EFSA) has mentioned, the recommended daily intake of copper is 1.3 mg for women and 1.6 mg for men over 18 years old^10^.

Several separation approaches have been developed for copper preconcentration to enhance the selectivity and sensitivity of analytical techniques. These approaches include Adsorption^11^, ion-exchange separation^12^, liquid-liquid extraction (LLE)^13^, gravity separation^14^, and cloud point extraction (CPE)^15–18^. Among the aforementioned techniques, Cloud point extraction has several advantages: it is rapid, simple to use, and environmentally friendly^19^, and it employs non-toxic surfactants^20–22^. The CPE procedure has been widely employed for the separation, purification, and pre-concentration of various substances, including organic and inorganic compounds, in water, food, pharmaceutical, and biological samples^23^. To overcome the main limitations of the traditional CPE, the RS-CPE approach was developed. Traditional CPE requires high energy use and long processing times. RS-CPE does not require heating or extended incubation periods. Additionally, RS-CPE employs a synergistic interaction that markedly accelerates cloud formation and improves extraction efficiency. RS-CPE enables faster extraction under mild temperature conditions, thereby reducing energy consumption and increasing analytical throughput. So, RS-CPE is considered a greener and more efficient alternative to traditional CPE^24^. For copper concentration determination, several spectroscopy methods have been utilized, including FAAS ‘’flame atomic absorption spectrometry’’, GFAAS ‘’graphite furnace atomic absorption spectrometry, and ICP-OES ‘’inductively coupled plasma / optical emission spectroscopy’’^25–27^. The ICP-OES technique was used for determining the Cu^2+^ concentration after the preconcentration step employing the CPE technique^28^.

Aryl hydrazones have gained considerable interest from chemists and researchers as potential candidates in analytical, coordination, pharmaceutical, and medicinal chemistry^29–32^. The merits of aryl hydrazone hybrids are attributed to their versatile and fascinating structural features, characteristic properties, and pervasiveness in biological activities, including anti-inflammatory, antifungal, antimicrobial, antibacterial, neuroprotective, pesticidal, and herbicidal effects^33,34^. Additionally, triazole-based hydrazones are considered one of the most effective hydrazine classes having tolerant fluorescence and spectrometric properties^35–38^. Furthermore, triazole hydrazone scaffolds are considered multidentate ligands with excellent coordination chemistry, featuring multiple free donor atoms that can be utilized in molecular switches, metallo-assemblies, and the synthesis of stable chemosensors for the hydrolysis process^39–41^. Interestingly, they have demonstrated a highly selective and sensitive ability to detect trace heavy metal cations (Cu^2+^, Zn^2+^, Hg^2+^, etc.), anions (F^−^, CN^−^, P_2_O_7_^4−^, etc.), and poisonous fumes, accommodating their coordination geometry^42,43^.

To the best of our knowledge, applying the CPE/ICP-OES and RS-CPE/ICP-OES techniques for Cu^2+^ removal using a 3-((4-amino-5-mercapto-4 H-1,2,4-triazol-3-yl)diazenyl)-1 H-indol-2-ol Schiff base ligand (HIT) and TX-114 surfactant has not been reported in the literature.

Based upon the aforementioned information, the objectives of the present study can be illustrated in the following points: (i) To prepare the triazole derivative Shiff base (HIT) and the copper-triazole derivative Schiff base nanocomplex (HIT-Cu^2+^); (ii) To characterize the HIT Schiff base and HIT-Cu^2+^ nanocomplex using FTIR, UV-vis, and TEM techniques; (iii) To preconcentrate and determine the Cu^2+^ in several environmental water samples applying CPE/ICP-OES and RS-CPE/ICP-OES techniques utilizing HIT, which acted as a chelating ligand in the presence of TX-114 non-ionic surfactant; (iv) To optimize the investigated parameters that may affect the Cu^2+^ preconcentration, such as the solution pH, Cu^2+^ and HIT concentration, the surface-active agent (TX-114) concentration, the influence of different types of surface-active agents, temperature, centrifugation time and rate, foreign ions influence, and solution volume; (v) To evaluate the greenness of the applied methods using AGREE and BAGI methodologies; (vi) To predict the HIT Schiff base and HIT-Cu^2+^ nanocomplex toxicity; (vii) To study the separation and measurement of Cu^2+^ in various water and pharmaceutical samples utilizing the HIT Schiff base.

## Experimental

### Reagents and solutions

In this investigation, the water utilized was doubly distilled. Additionally, all the chemicals used were of analytical reagent grade: copper(II) chloride, hydrated CuCl_2_·6H_2_O, purchased from Merck. Different stock solutions were prepared: a 1 × 10^− 3^ mol.L^-1^ Cu^2+^ stock solution was obtained by dissolving 0.0242 g CuCl_2_.6H_2_O in 100 mL DDW in the presence of 1 mL HCl (conc). Stock solution of the Schiff base (HIT) (1 × 10^− 3^ mol.L^-1^) was prepared by dissolving 0.02573 g (HIT) in 100 mL of ethanol. The TX-114 was utilized without additional purification. A TX-114 stock solution of 1% (v/v) was obtained by putting 1 mL of it in 5 mL of ethanol and then completing the volume to 100 mL with DDW.

### Instruments

Using KBr tablets and a JASCO FT/IR-460 spectrophotometer at room temperature, the FT-IR spectra for HIT, HIT-Cu^2+^, and HIT-Cu^2+^ in a rich phase were reported in the 400–4000 cm^− 1^ range. In a cell (1 cm, quartz), a PerkinElmer 550 Spectrophotometer was utilized for the UV-Vis spectra estimation of the HIT Schiff base and the HIT-Cu^2+^ complex before and after the preconcentration in a wavelength range (190–900 nm). The Cu^2+^ concentration was determined using a Varian ICP-OES Vista Pro (CCD Simultaneous). The particle size and morphology of the HIT Schiff base and HIT-Cu^2+^ complex were measured using Transmittance Electron Microscopy, TEM (JEOL JEM-2100).

### Preparations

#### Preparation of 3-(2-(4-amino-5-mercapto-4 H-1,2,4-triazol-3-yl)hydrazono) indolin-2-one (HIT)

**Fig. 1:**
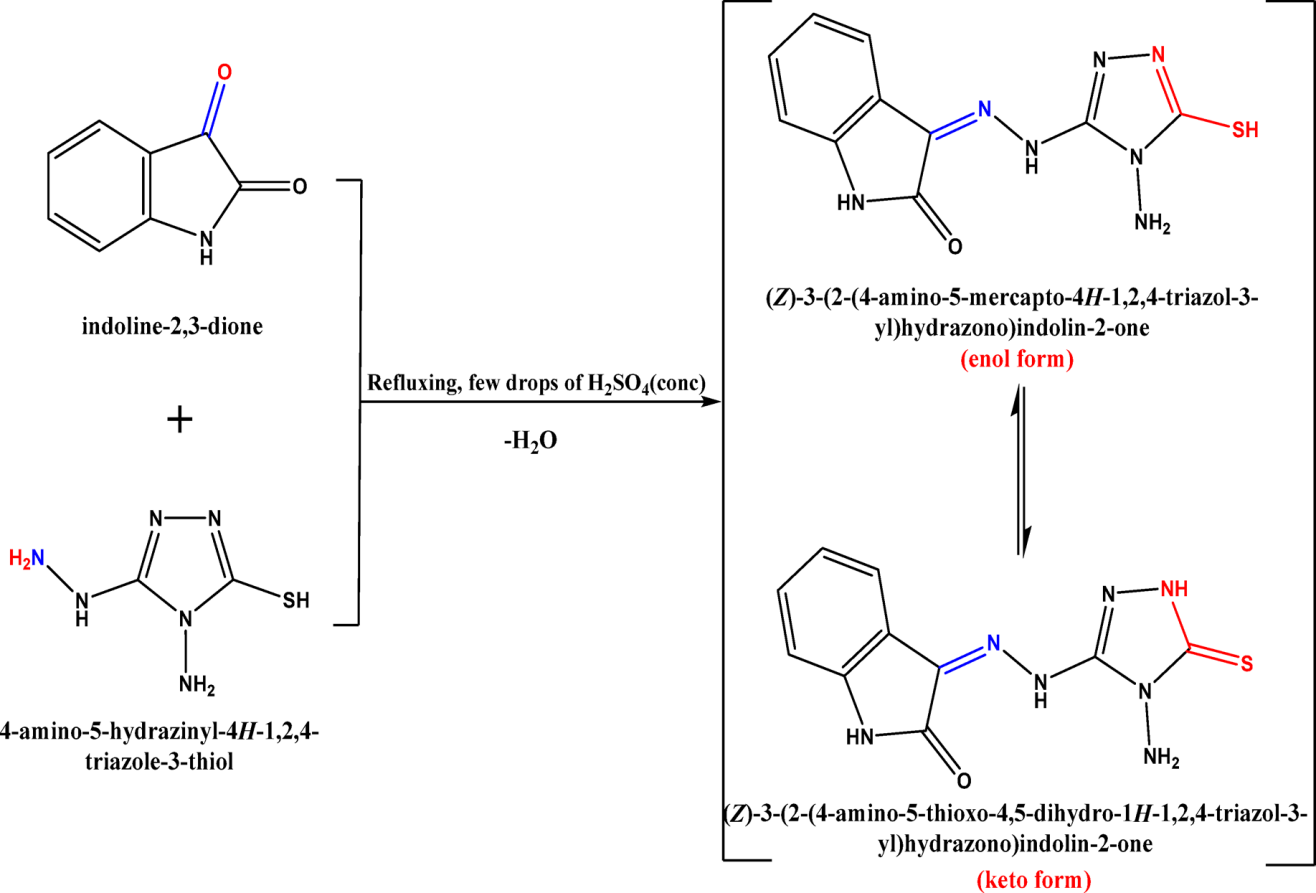
Synthesis of Schiff base ligand (HIT).

As presented in Fig. 1, 3-(2-(4-amino-5-mercapto-4 H-1,2,4-triazol-3-yl)hydrazono)indolin-2-one (HIT) was prepared as follows: at first, 1.0 mmol of the 4-amino-5-hydrazinyl-4 H-1,2,4-triazole-3-thiol compound was mixed with 1.0 mmol of isatin (indoline-2,3-dione) in 25 mL of methanol with the addition of a few drops of Conc. H_2_SO_4_ was then added to the mixture, which was allowed to reflux for 5 min. The reaction ending was observed through TLC. The resultant precipitate was obtained on heating. It was filtered and dried. Finally, the obtained precipitate was washed with hot methanol without any further purification^44^.

#### Synthesis of HIT-Cu^2+^ nanocomplex

**Fig. 2:**
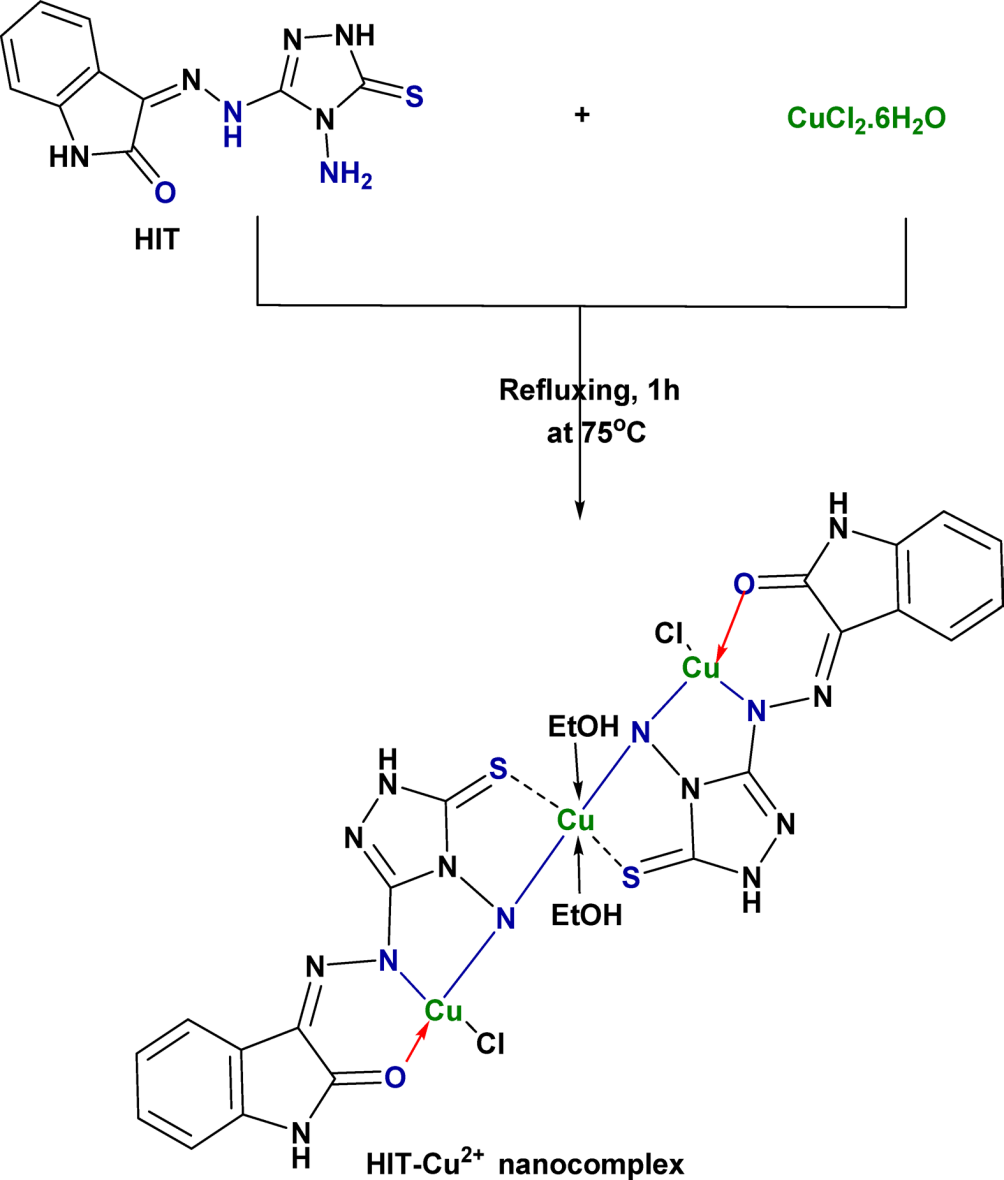
The proposed mechanism of the synthesis of the HIT-Cu^2+^ complex.

The HIT-Cu^2+^ nanocomplex, Fig. 2, was prepared by refluxing CuCl_2_.6H_2_O (1.0 mol.L^− 1^, 0.2573 g) dissolved in 10 mL of EtOH for 1 h at 75 °C. Once the Schiff base (HIT) was added to the Cu^2+^ solution, a color shift was observed as the solution became dark brown. As the reaction proceeded, a brownish precipitate of the HIT-Cu^2+^ complex was formed. Finally, the formed HIT-Cu^2+^ precipitate was centrifuged, washed numerous times, and dried under a vacuum atmosphere.

#### General procedures of CPE and RS-CPE

##### Conventional CPE

For Cu^2+^ separation and determination, a sample solution containing 3.147 × 10^− 5^ mol.L^− 1^ of Cu^2+^ was transferred to a 15 mL centrifuge tube containing 2 mL acetate buffer (0.1 mol.L^− 1^, pH 5). Then, 2.098 × 10^− 5^ mol.L^− 1^ of HIT and 1 mL of TX-114 (0.04%, V/V) were added, and the solution was diluted to 10 mL with DDW. After that, the prepared tube is placed in a water bath for 15 min at 50 °C to reach equilibration. Subsequently, the centrifuge tube was placed in an ice bath for 15 min. Then, the centrifugation step, which lasted 10 min for the sample, occurred at a speed of 3000 rpm. Finally, the TX-114-rich phase will settle as a brown spot at the bottom of the centrifuge tube, and the aqueous layer above can then be easily removed by aspiration. To determine the Cu^2+^ concentration, the TX-114-rich phase was eluted utilizing 1 mol.L^− 1^ HNO_3_ and introduced to ICP-OES. The CPE separation efficiency (E%) was estimated in the TX-114-rich phase, as presented in Eq. (1).

1 $$\:E\left(\%\right)=\frac{{C}_{i}}{{C}_{f}}*100$$

C_i_ and C_f_ were the initial Cu^2+^ concentration in the mother solution before the CPE step and the final concentration after the CPE step in the TX-114-rich phase.

##### RS-CPE

**Fig. 3:**
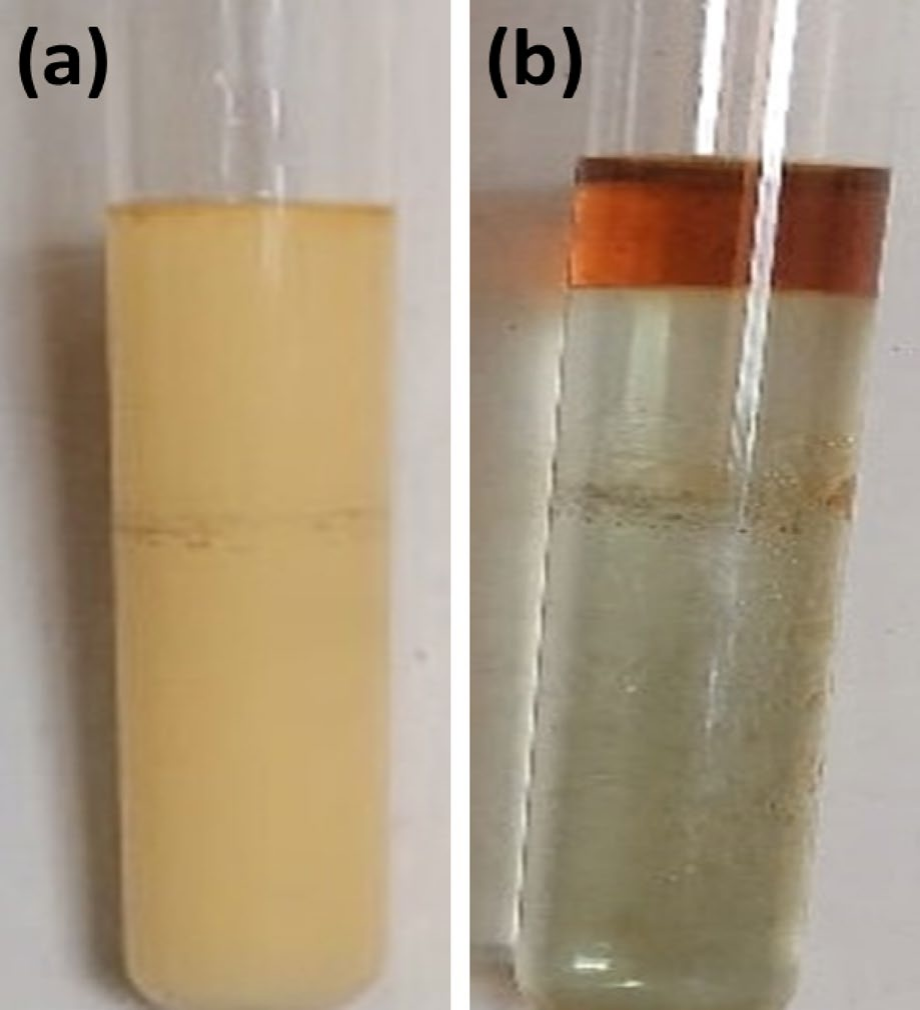
(**a**) Dispersed solution of HIT-Cu^2+^ complex in decanol and (**b**) HIT-Cu^2+^ in TX-114-rich phase with decanol after centrifugation.

The RS-CPE process is a rapid process that does not require any heating. The RS-CPE experiment was obtained as follows: a 2.098 × 10^− 5^ mol.L^-1^ of HIT solution was added to a solution containing 3.147 × 10^− 5^ mol.L^-1^ of Cu^2+^, which was transferred to a 15 mL centrifuge tube containing 2 mL acetate buffer (0.1 mol.L^-1^, pH 5). Then, the proper amounts of TX-114 (0.04%, V/V) and 2 mL of decanol were added to the tube. After shaking the tube, the solution became turbid, as shown in Fig. 3a, and micelles formed due to the effect of decanol. The extraction was accomplished rapidly during the shaking process, lasting approximately 1 min. The HIT-Cu^2+^ complex was captured by the TX-114 micelle and dispersed in decanol. Then, the centrifugation step, lasting 5 min for the sample, occurred at a speed of 3000 rpm. As presented in Fig. 3b, the turbid yellow solution became clear after centrifugation, and the supernatant, which was rich in TX-114 and decanol, resulted in a deep brown color. To determine the Cu^2+^ concentration, the TX-114-rich phase was eluted utilizing 1 mol.L^-1^ HNO_3_ and introduced to ICP-OES. The CPE separation efficiency (E%) was estimated in the TX-114-rich phase, as presented in Eq. (1).

### Application

#### Analysis of water samples

The applicability of the HIT Schiff base was assessed utilizing three real water samples. These samples were collected from various locations, including seawater from Ras El-Bar, tap water from Mansoura City, and Nile water from the Nile River. The Investigated water samples were pre-treated before the analysis, as they were filtered. In addition, the pH was adjusted to 1 by adding a few drops of HCl(conc). Various concentrations of Cu^2+^ were spiked into 25 mL aliquots of the investigated water samples; 2.098 × 10^− 5^ mol.L^− 1^ HIT and 1 mL of 0.04% TX-114 were added. The same previous steps of CPE and ICP determination were performed.

#### Pharmaceutical samples

Two copper-containing vitamin tablets were utilized for the Cu^2+^ analysis by applying the HIT Schiff base. The investigated tablets were solubilized using the following procedure: Each tablet was treated separately with HNO_3_ (conc) at 50 °C. Then each tablet residue was cooled, adding 1:1 HNO_3_ with a gradual temperature increase to about 300 °C for two hours to yield a dry mass. Finally, the dry mass of each tablet was dissolved in distilled H_2_O^45^.

## Results and discussion

### Characterization

#### FT-IR spectra

**Fig. 4:**
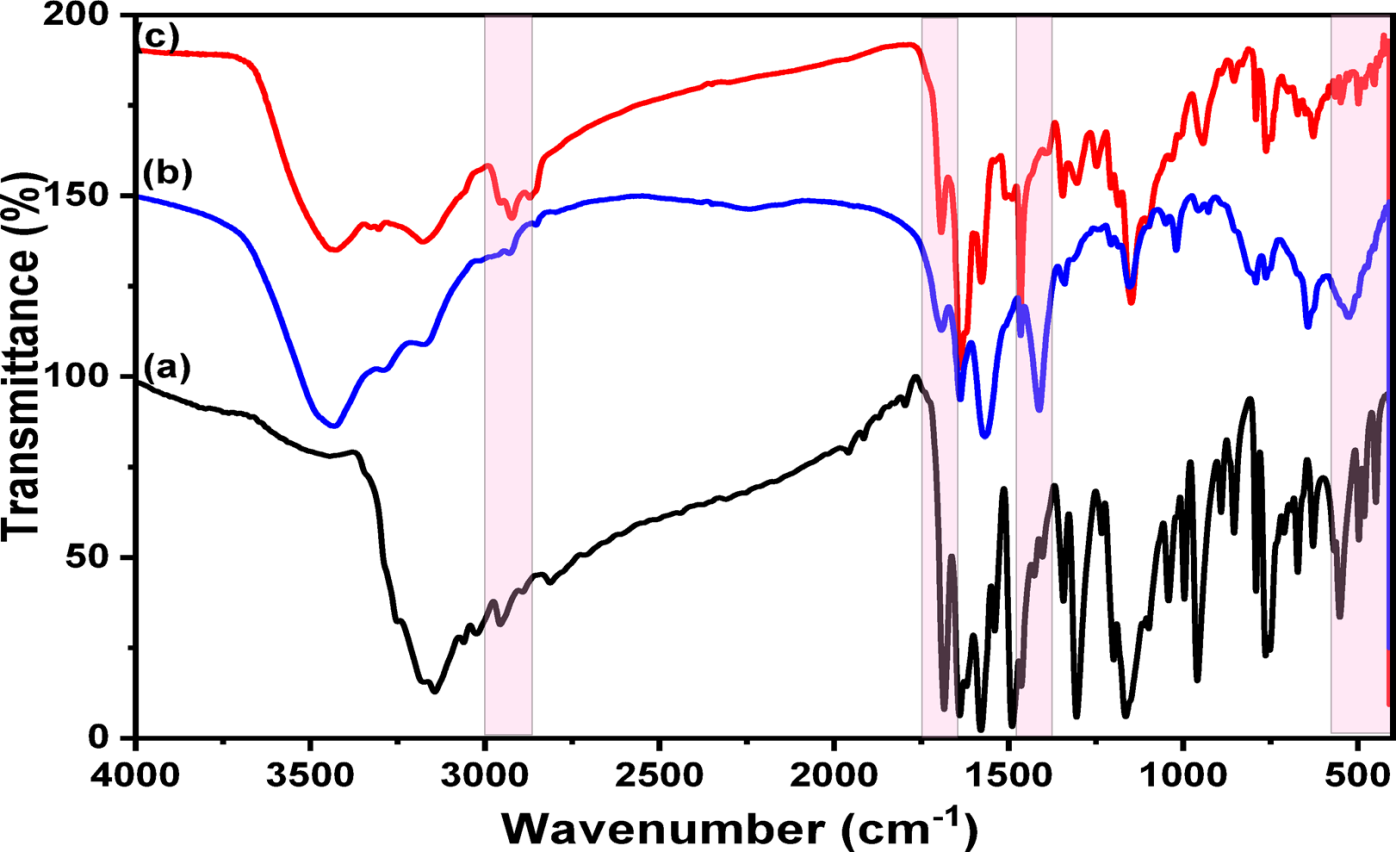
The FTIR spectra of (**a**) HIT Schiff base, (**b**) HIT-Cu^2+^ complex, and (**c**) HIT-Cu^2+^ complex in the organic layer.

Figure 4a–c demonstrates the FTIR spectra of the ligand HIT, the HIT-Cu^2+^ complex, and the HIT-Cu^2+^ complex in the organic layer. For the HIT ligand (Fig. 4a), the band at about 3500 cm^− 1^ may be attributed to the hydroxyl groups (OH). The splitting pattern peak that appeared at (3184 –3144 cm^− 1^) is assigned to the NH_2_. The bands at 3241, 3061, and 2811 cm^− 1^ may be attributed to the 2-ry amine NH, C-H of the alkene, and S-H, respectively. The C=O, C = N, C-N, and C-S bands appeared at about 1686 cm^− 1^, 1640 cm^− 1^, 1301 cm^− 1^, and 630 cm^− 1^, respectively^44^.

For the HIT-Cu^2+^ complex (Fig. 4b), it was observed that the disappearance of the NH_2_ peak and the shift of the C = O band from 1684 cm^− 1^ to 1697 cm^− 1^. Further, the appearance of a new peak at about 1410 cm^− 1^ may be attributed to the C = S peak. Besides, the whole disappearance of the S-H peak demonstrates the triazole tautomerism occurrence before the complexation step, as presented in Fig. (1)^46^. New peaks at 533 cm^− 1^ and 452 cm^− 1^ are assigned to Cu-N and Cu-S, respectively^47–49^. It was concluded that the Cu^2+^ complexation with the HIT Schiff base is formed through C=O and NH to form a stable six-membered complex and from NH_2_ and C=S to form a stable five-membered complex^46^. The HIT-Cu^2+^ complex in the organic layer (Fig. 4c) showed CH_3_–CH_2_ characteristic peaks of TX-114 at about 2923 cm^− 1^ and 2866 cm^− 1^, respectively.

#### UV-vis spectra

**Fig. 5:**
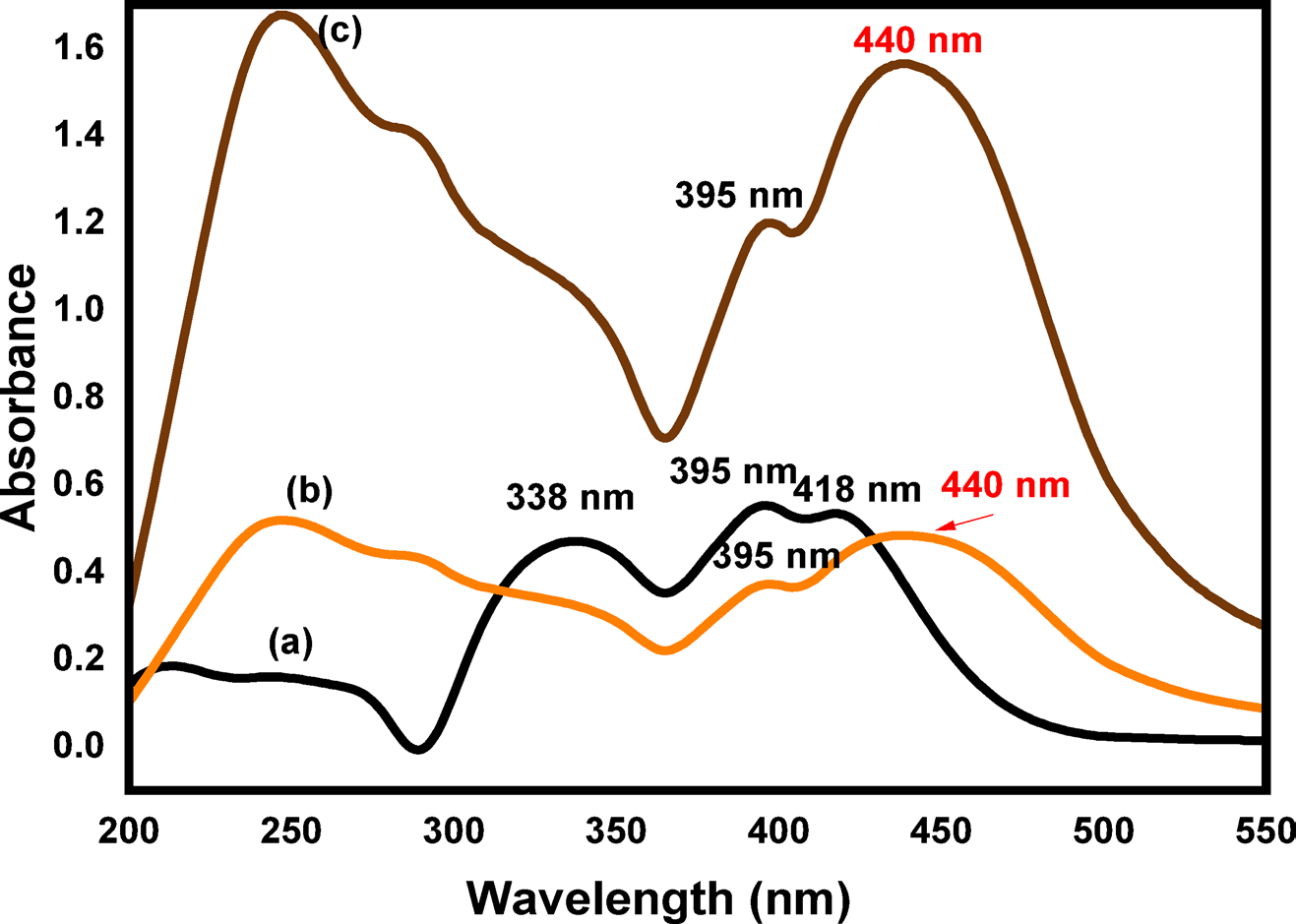
The electronic absorption spectra of (**a**) Schiff base ligand (HIT), (**b**) Copper complex (HIT-Cu^2+^) in aqueous solution, and (**c**) Copper complex (HIT-Cu^2+^) in TX-114.

Figure 5 indicates the electronic absorption spectra of HIT and HIT-Cu^2+^ in the aqueous solution and in the TX-114-rich phase. As shown in Fig. 5a, the HIT exhibits three characteristic bands at 338, 395, and 418 nm. As observed, there is a significant difference in the absorption spectra of the HIT and HIT-Cu^2+^ complex. Figure 5b, the absorption spectra of the HIT-Cu^2+^ complex, which exhibit the appearance of a new peak at 440 nm and the disappearance of the HIT characteristic peak at 338 nm, and the peak at 418 nm is red-shifted to 440 nm. After the preconcentration step utilizing TX-114, the absorbance spectrum is presented in (Fig. 5c). This demonstrates the effectiveness of the preconcentration step as the HIT-Cu^2+^ absorbance in the Triton X-114 system is about four times greater than that of HIT-Cu^2+^ in an aqueous solution^50,51^.

#### Stoichiometric ratio

The Cu²⁺ to HIT Schiff base stoichiometric ratio was estimated using the continuous variation (Job’s method), as well as the HIT concentration parameter. In this approach, solutions of the Cu²⁺ and the HIT Schiff base at the same concentration were mixed in varying volumes while keeping the total concentration constant. The maximum absorbance observed corresponds to the stoichiometric ratio of the metal-ligand complex, which in this case indicates a ratio of 3:2 (Cu²⁺ : HIT), Fig. 1S.

#### Elemental analysis

The CHNS analysis of the HIT Schiff base and the HIT-Cu^2+^ complex is shown in Table 1. It was observed that the results calculated for the proposed formula agreed with those obtained.

**Table 1:** Elemental analysis of HIT schiff base and HIT-Cu^2+^ complex.

Samples	C (%)		H (%)		N (%)		S (%)	
	Calculated	Obtained	Calculated	Obtained	Calculated	Obtained	Calculated	Obtained
HIT	43.63	43.14	3.3	3.41	35.62	35.28	11.65	11.43
HIT-Cu^2+^	30.68	30.94	1.96	2.03	23.85	24.07	7.8	7.98

#### Transmission electron microscope (TEM)

**Fig. 6:**
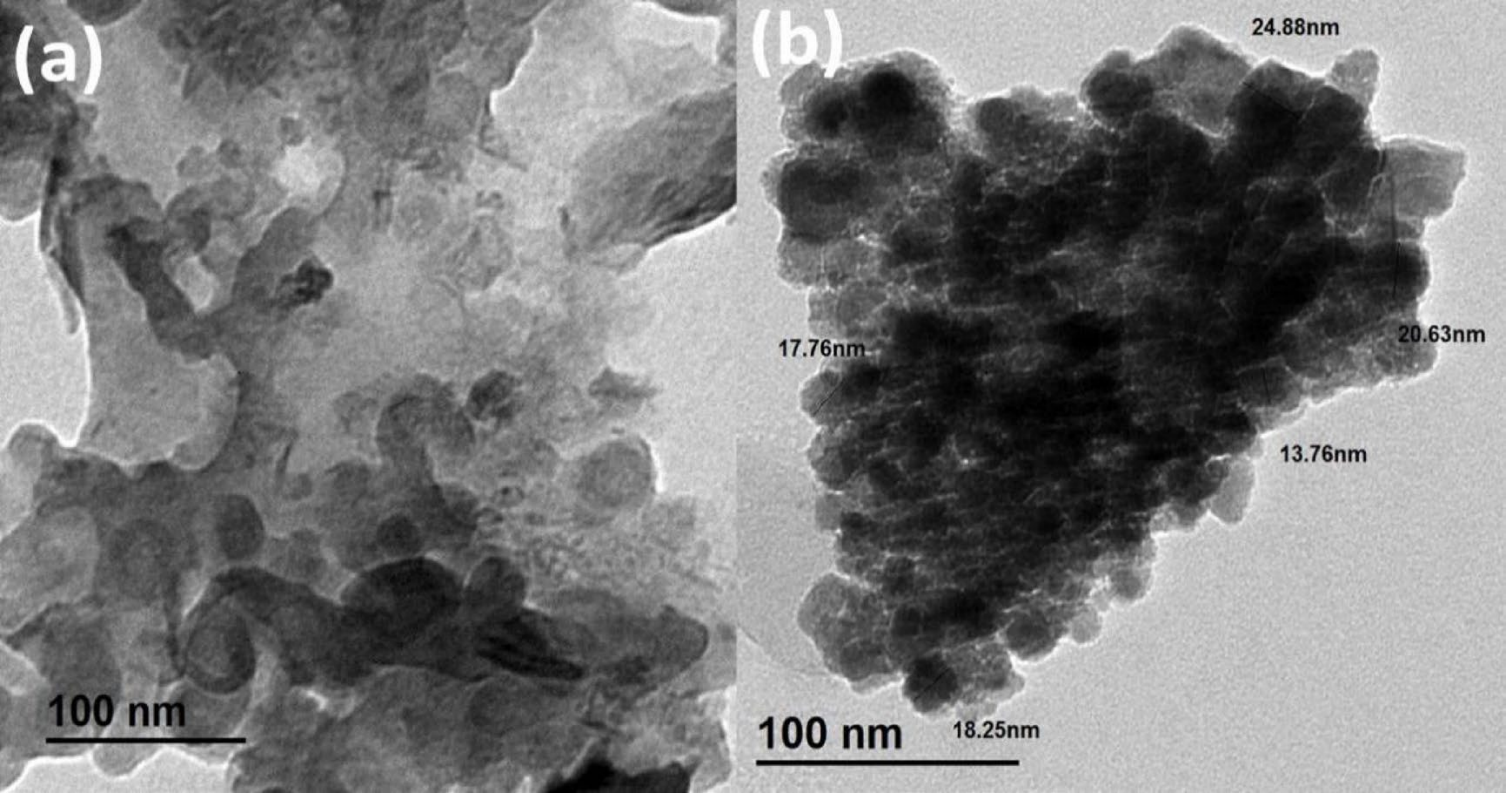
TEM micrographs of (**a**) HIT Schiff base and (**b**) HIT-Cu^2+^ complex.

TEM investigation characterized the morphology, size, and shape of HIT and HIT-Cu^2+^ compounds, as presented in Fig. 6. As shown in Fig. 6a, the HIT morphology is irregular, with large aggregated particles that have inadequately distinct boundaries, revealing the absence of a crystalline nanostructure. Meanwhile, the HIT-Cu^2+^ complex TEM micrograph, Fig. 6b, displays uniformly distributed nanoparticles with a spherical shape. The HIT-Cu^2+^ particle size ranges from 13 to 25 nm, indicating that the complexation process was successful and resulted in the formation of nanoscale structures. The observed clustering of nanoparticles is likely due to their high surface energy and intense interactions with each other. These results support transitioning from an amorphous ligand phase to well-defined nanostructured complexes.

#### Digital photographs of CPE

**Fig. 7:**
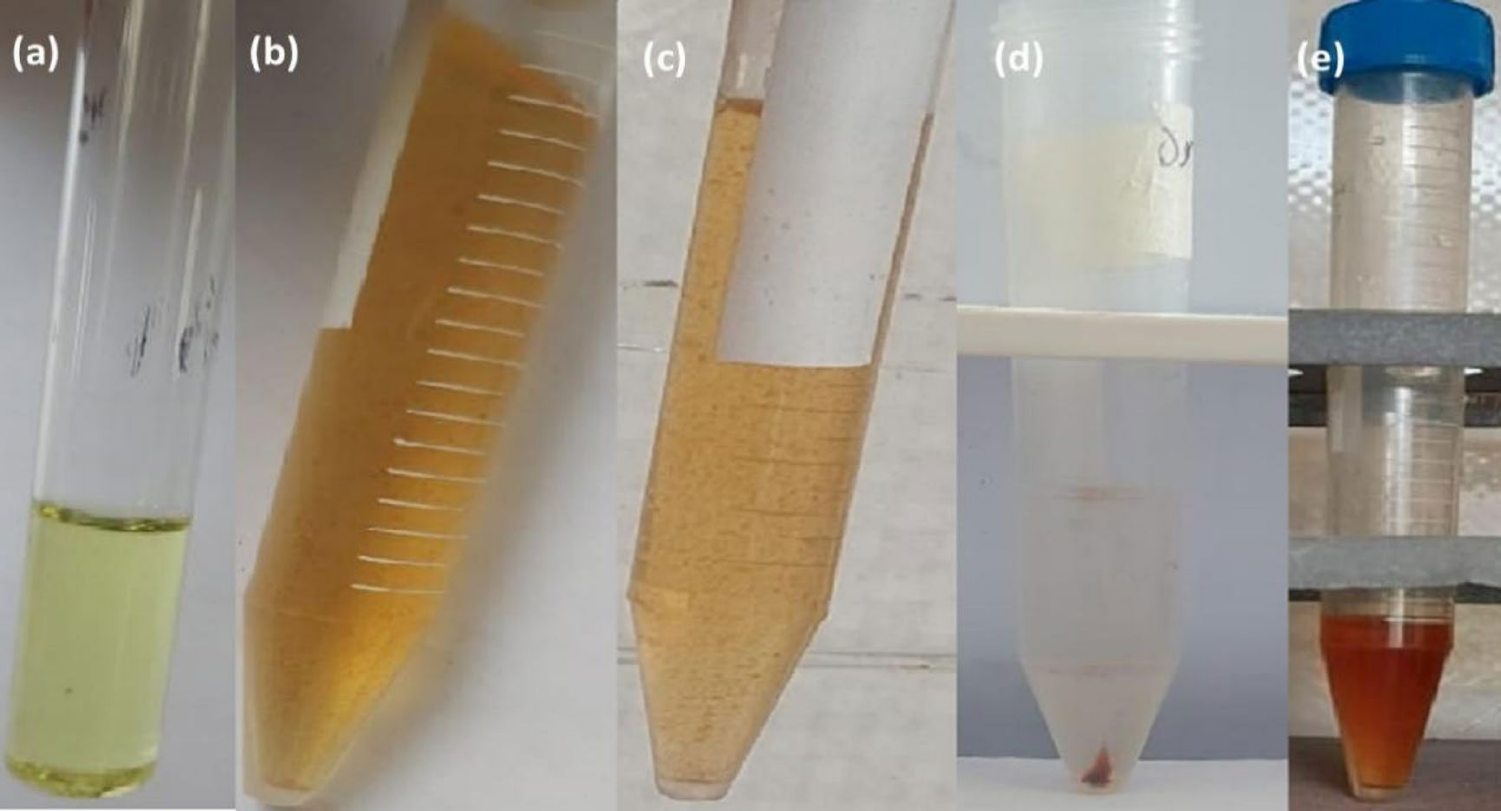
(**a**) solution of HIT, (**b**) HIT-Cu^2+^ nanocomplex in solution, (**c**) HIT-Cu^2+^ after hot water bath, (**d**) the spot formation after centrifugation step, and (**e**) the spot dissolved in ethanol.

Figure 7 represents the digital photographs of HIT and the steps of CPE of the HIT-Cu^2+^ complex. Figure 7a represents a clear yellow solution of the HIT Schiff base. Figure 7b, c show the liquid complex before and after the hot and ice water baths, respectively. As can be seen, the color shifted to orange after the HIT-Cu^2+^ complex formation. It was observed that the homogenous solution in Fig. 7b turned into a turbid solution (Fig. 7c) after the hot water bath. Figure 7d shows the cloud point spot formation after the centrifugation step and the clear colorless solution, proving the complete extraction of the complex. Meanwhile, Fig. 7e represents the spot after it has dissolved in ethanol.

### Effect of experimental variables

#### Influence of pH

**Fig. 8:**
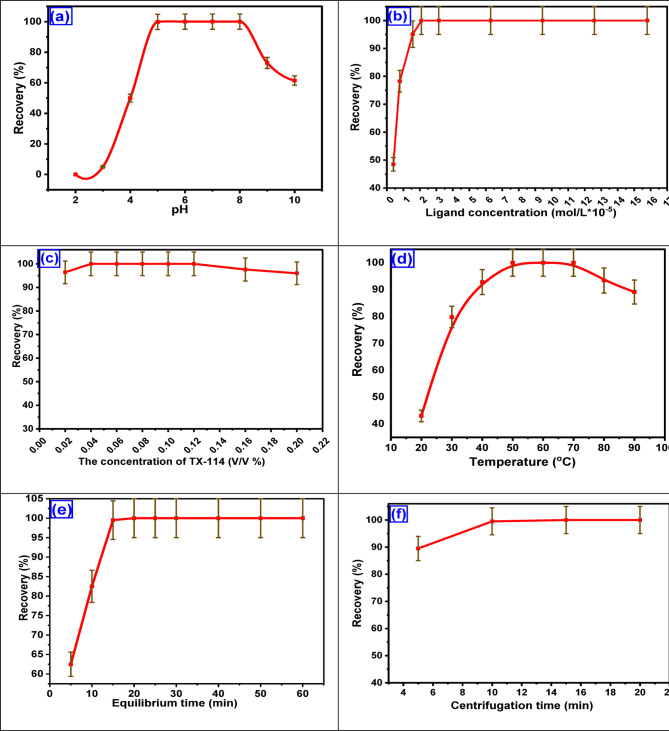
Parameters affecting the CPE/ICP-OES of the Cu^2+^ (**a**) pH of 3.147 × 10^− 5^ mol.L^− 1^ of Cu^2+^, 9.44 × 10^− 5^ mol.L^− 1^ of HIT, and 0.1% (v/v) TX-114, (**b**) HIT Schiff base ligand concentration of Cu^2+^ 3.147 × 10^− 5^ mol.L^− 1^ using 0.1% (v/v) TX-114 at pH 5, (**c**) TX-114 concentration of 3.147 × 10^− 5^ mol.L^− 1^ Cu^2+^ at pH 5 using HIT (2.098 × 10^− 5^ mol.L^− 1^), (**d**) equilibrium temperature, (**e**) equilibrium time of 3.147 × 10^− 5^ mol.L^− 1^ Cu^2+^ at pH 5 using HIT (2.098 × 10^− 5^ mol.L^− 1^) and 0.04% (V/V) TX-114, (**f**) centrifugation time of 3.147 × 10^− 5^ mol.L^− 1^ Cu^2+^ at pH 5 using HIT (2.098 × 10^− 5^ mol.L^− 1^) and 0.04% (v/v) TX-114 at 50 °C.

pH factor is a crucial parameter in the metal chelation process and the production of extractable species from ionic analytes via the Schiff base HIT. The effect of pH on the extraction of Cu^2+^ was investigated in the pH range (2–6) using 3.147 × 10^− 5^ mol.L^− 1^ of Cu^2+^ with 9.44 × 10^− 5^ mol.L^− 1^ of HIT and 0.1% (v/v) Triton X-114. The results are presented in Fig. 8a. The maximum absorbance occurred at a pH of 5. At pH lower than 5.0, Cu^2+^ recoveries decreased. This may be due to the competition between Cu^2+^ and protons, resulting in lower metal chelation with HIT. Then, the Cu^2+^ recovery remained constant as the pH increased from 5 to 8. With a further increase in pH, the Cu^2+^ recovery decreases, and this is attributed to the formation of the hydroxide form of copper. Hence, the following work was performed at pH five by adding the acetate buffer solution.

#### Influence of concentration of HIT ligand and Cu^2+^

The influence of HIT concentration on the Cu^2+^ extraction was evaluated in the range of 0.4 × 10^− 5^ to 15.7 × 10^− 5^ using an appropriate concentration of Cu^2+^ 3.147 × 10^− 5^ mol.L^− 1^ and 0.04% (v/v) TX-114 at pH 5. Figure 8b showed that the extraction efficiency increased as the HIT concentration increased from 0.4 × 10^− 5^ to 1.57 × 10^− 5^ mol.L^− 1^. The maximum extraction efficiency of Cu^2+^ was found at HIT concentration (2.098 × 10^− 5^ mol.L^− 1^) at a ratio of 2:3 (HIT: Cu^2+^). Increasing HIT concentration above 2.098 × 10^− 5^ mol.L^− 1^ showed no effect on the Cu^2+^ extraction efficiency. Therefore, a concentration of 2.098 × 10^− 5^ mol.L^− 1^ is used in the subsequent experiments.

#### Influence of different types of surfactants

Three surfactant were utilized to evaluate the maximum extraction of the HIT-Cu^2+^ complex. The investigation occurred through the interaction between the HIT-Cu^2+^ complex and the hydrophobic surfactant layer. The extraction efficiency (E, (%)) was investigated utilizing two types of surfactants: neutral (Triton X-100 and Triton X-114) and cationic (cetyltrimethylammonium bromide (CTAB)). As shown in Table 2, the maximum extraction of the HIT-Cu^2+^ complex was achieved by using the neutral surfactant TX-114. So, Triton X-114 was chosen for this work.

**Table 2:** Effect of different types of surfactants on CPE/ICP-OES of Cu^2+^ using (3.147 × 10^− 5^ mol.L^− 1^ analyte at pH 5 in the presence of 2.098 × 10^− 5^ mol.L^− 1^ HIT), (*n* = 3).

Surfactant	E (%)
TX-100	94
TX-114	100
CTAB	68

#### Influence of TX-114 concentration

TX-114 was utilized as it achieved 100% extraction for the HIT-Cu^2+^ complex. In addition, TX-114 has several advantages, including being commercially available in a highly refined, homogeneous form, being inexpensive, and exhibiting acceptable toxicological characteristics. Due to the high density of the surfactant-rich phase, the centrifugation step is easily achieved. The effect of TX-114 concentration on the HIT-Cu^2+^ complex was studied in the 0.02–0.2% (v/v) range. As shown in Fig. 8c, the Cu^2+^ extraction increased as the TX-114 concentration increased from 0.02% to 0.04%, reaching a maximum extraction of Cu^2+^ at a TX-114 concentration of 0.04%. With the increase of TX-114 concentration from 0.04% to 0.1%, the Cu^2+^ extraction remained constant. Hence, 0.04% (v/v) of TX-114 was maintained at a constant level throughout all subsequent experiments.

#### Effect of equilibrium temperature and time

The equilibrium temperature parameter was investigated in the range of (20–90 °C). The data in Fig. 8d show that the highest recovery was achieved between 50 and 70 °C. After 70 °C, the recovery decreased as the complex broke down at higher temperatures. Moreover, the incubation time parameter was investigated within the range of (5–60 min). As shown in Fig. 8e, 15 min is an adequate incubation time for the maximum recovery of Cu^2+^. The findings demonstrate that an incubation time of 15 min and a temperature of 50 °C were selected for further investigation^52^.

#### Influence of centrifugation time and rate

The centrifugation time and rate parameters affecting the recovery of Cu^2+^ extraction were investigated to achieve the optimum separation conditions. The centrifugation rate and time parameters were investigated in (500–4000 rpm) and (5–20 min). After 10 min, the surfactant-rich phase was separated (Fig. 8f). Therefore, the applied centrifugation time and rate in the subsequent experiments were 10 min and 3000 rpm, respectively.

#### Influence of volume

**Fig. 9:**
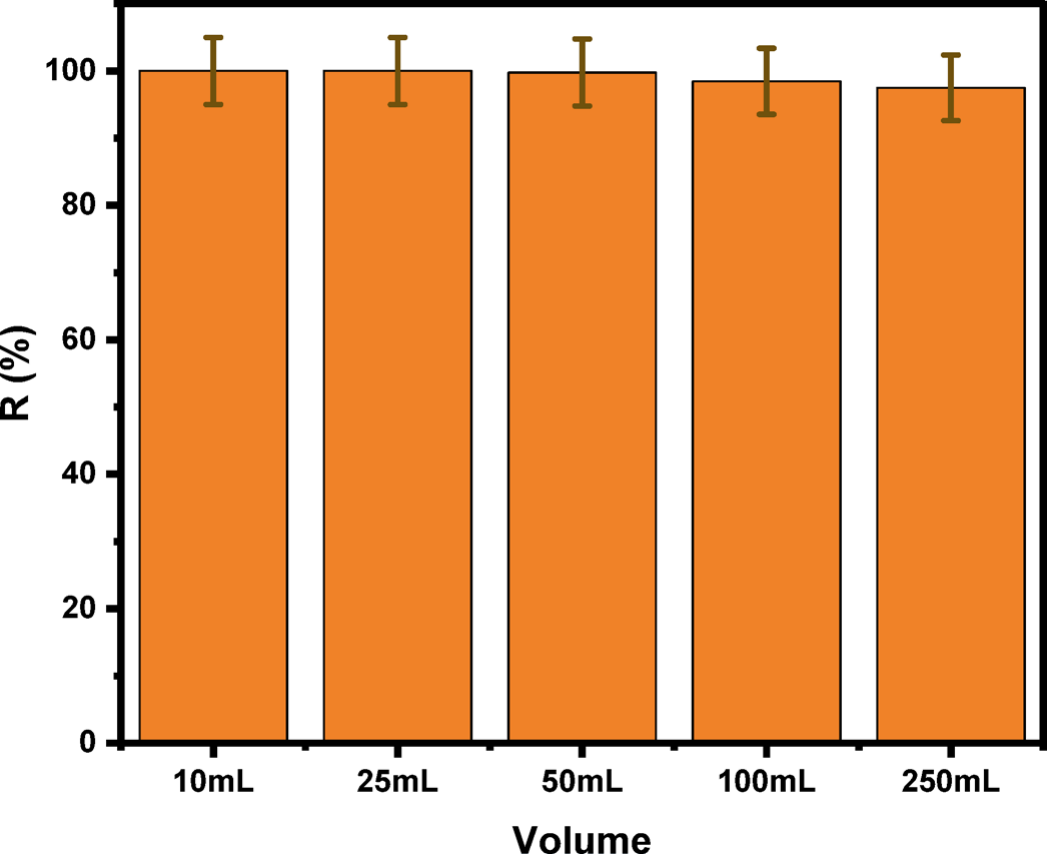
Effect of volume on CPE/ICP-OES of 3.147 × 10^− 5^ mol.L^− 1^ Cu^2+^ at pH 5 using HIT (2.098 × 10^− 5^ mol.L^− 1^) and 0.04% (v/v) TX-114 at 50 °C.

A series of experiments was conducted under the recommended conditions to extract 3.147 × 10^− 5^ mol.L^− 1^ of Cu^2+^ from various volumes. Thus, various volumes of a water sample, ranging from 10 to 250 mL, and a constant TX-114 surfactant concentration of 0.04% were chosen. CPE efficiency was not significantly affected (R(%) > 97%). The extraction solutions before and after phase separation volumes were measured, and the enrichment (preconcentration) factor was calculated based on the surfactant-rich phase ([V]s) and aqueous phase ([V]a) volumes applying Eq. (2). Therefore, this study has an enrichment factor of 125 for the investigated analyte (Fig. 9)^51^.

2 $$\:\mathrm{E}\mathrm{n}\mathrm{r}\mathrm{i}\mathrm{c}\mathrm{h}\mathrm{m}\mathrm{e}\mathrm{n}\mathrm{t}\:\mathrm{f}\mathrm{a}\mathrm{c}\mathrm{t}\mathrm{o}\mathrm{r}=\frac{{\left[v\right]}_{a}}{{\left[v\right]}_{b}}$$

#### Influence of different eluting solvents

At the end of the CPE methodology steps (centrifugation), a viscous TX-114-rich phase spot was formed that must be diluted before aspiration to the ICP-OES. Therefore, various eluents, including ethanol, methanol, HNO_3_, and HCl, were investigated to determine the optimal one based on analytical signal sensitivity. It was observed that the 1 mol.L^− 1^ HNO_3_ achieved the maximal signal, thus selecting it as the optimal eluting solvent.

#### Influence of added electrolyte

Salt addition is often employed for ionic strength adjustment as it enhances extraction efficiency and decreases the detection limit. The KCl concentration influences the HIT-Cu^2+^ separation, which was investigated by varying its concentration from 0.01 mol.L^− 1^ to 0.1 mol.L^− 1^. It was shown that the HIT-Cu^2+^ extraction efficiency increased with KCl concentration, rising from 0.01 mol.L^− 1^ to 0.05 mol.L^− 1^, and then decreased above 0.05 mol.L^− 1^ of KCl concentration. The Cu^2+^ separation efficiency enhancement may be attributed to increased hydrophobic interaction between the TX-114 micelles and Cu^2+^. As elevated KCl concentrations increase the water drops’ density, it may lead to the phase separation disturbance.

#### Foreign ions

The influence of foreign ions on Cu^2+^ recovery using the HIT Schiff base was investigated under optimal conditions, and the results are presented in Table 3. It was obtained by adding known concentrations of the investigated ions to a Cu^2+^-containing aqueous solution, using the investigated approach. The tolerance level was described as the maximum amount of the foreign species that produced an error of ± 5% in Cu^2+^ recovery. As shown in Table 3, the results demonstrated that some ions have no considerable effect on the Cu^2+^ response, while others have acceptable limits of interference with Cu^2+^^51^.

**Table 3:** Tolerance limits for the determination of 2 µg.mL^− 1^ Cu^2+^.

Foreign ions	Concentration (µg.mL^− 1^)	*R*%
		Cu^2+^
Hg^2+^	2	99
Zn^2+^	2	98.2
Cd^2+^	1	96.7
Al^3+^	3	98.8
Mn^2+^	2	97.8
Co^2+^	2	99.1
Cr^3+^	3	99
NO_3_^−^	310	96.8
Cl^−^	2300	98.4

### Analytical figures of merit

At the investigated optimum conditions, the analytical figures of merit for the investigated methods, CPE and RS-CPE, were compared as presented in Table 4. Cu^2+^ calibration graphs with a correlation coefficient (r) value greater than 0.999 are linear in the 0.5–5 µg.mL^− 1^ range for both approaches. The detection limit was 0.016–0.018 µg.L^− 1^ (LOD = 3.3 SD/m), where SD and m represent the standard deviation of the blank and the slope of the calibration curve, respectively. The limit of quantification (LOQ = 3LOD) was 0.048 and 0.054 µg.L^− 1^ for the RS-CPE and CPE, respectively. The relative standard deviation was in the range of (0.255–0.270)% (*n* = 5).

**Table 4:** The analytical figures of merit of the CPE and RS-CPE methods.

Methodology	CPE	RS-CPE
Parameter	Cu^2+^	
Linear range (µg.mL^− 1^)	0.5-5	0.5-5
Calibration equation	y = 0.32749x- 0.00145	y = 0.32536x- 0.01386
SD	0.0018	0.0016
RSD % (*n* = 5)	0.270	0.255
LOD (µg.L^− 1^)	0.018	0.016
LOQ (µg.L^− 1^)	0.054	0.048
Enrichment factor	5	5
Correlation coefficient	0.9999	0.9997
Sample volume (mL)	10	10

### Application

#### In real water samples

The investigated approach for the Cu^2+^ determination using a CPE methodology was applied to various water samples, including tap, sea, and river water, to examine the approach’s validity and applicability. At a pH value of 5, various Cu^2+^ concentrations (1, 2, and 5 µg.mL^− 1^) were spiked to the investigated water samples. The formed spot was first diluted into 2 mL using ethanol and then measured using ICP-OES for Cu^2+^ analysis after CPE (as previously indicated). As shown in Table 5, the investigated approach attained recoveries higher than 97% with RSD less than 1% (*n* = 3). These findings suggest that this analytical approach could be used to detect Cu^2+^ in actual water samples.

**Table 5:** Determination of Cu^2+^ in natural water samples by CPE/ICP-OES using HIT (2.098 × 10^− 5^ mol.L^− 1^), 0.04% (V/V) TX-114 at pH 5.0 at 50 °C: (*n* = 3).

Water sample (Location)	Spiked (µg.mL^− 1^)	Recovered (µg.mL^− 1^)	Recovery (%)	RSD (%)
Tap water (Our lab)	**1**	0.98	98	0.298
	**2**	1.972	98.58	0.527
	**5**	4.963	99.26	0.154
Nile water (Mansoura city)	**1**	0.988	98.8	0.15
	**2**	1.97	98.7	0.7
	**5**	4.96	99.18	0.298
Seawater (Ras El-Bar city)	**1**	0.981	98.1	0.256
	**2**	1.955	97.75	0.18
	**5**	4.94	98.81	0.2

#### Pharmaceutical samples

To assess the applicability of the investigated approach, the CPE followed by ICP-OES determination was applied to determine Cu^2+^ in some multivitamin samples utilizing the HIT Schiff base. As shown in Table 6, the Cu^2+^ recovery from the examined pharmaceutical samples utilizing the HIT Schiff base through CPE technology was about 100%.

**Table 6:** Determination of Cu^2+^ in pharmaceutical samples by CPE/ICP-OES using HIT (2.098 × 10^− 5^ mol.L^− 1^), 0.04% (V/V) TX-114 at pH 5.0 at 50 °C: (*n* = 3).

Pharmaceutical sample	Spectrophotometry			Recovered (µg.mL^− 1^)	Recovery (%)	RSD (%)
	Observed (µg.mL^− 1^)		Prepared (µg.mL^− 1^)			
Vitastress	60	2		1.995	99.75	0.21
Totavit	18	2		2	100	0.13

### Performance of the azo-linked triazole (HIT) schiff base

The analytical performance of the current method has been compared with previously published studies that investigated Cu^2+^ preconcentration. As mentioned in the studies in Table 7, the current study evaluated precision and sensitivity, achieving lower values of RSD (%) and LOD of 0.27% and 0.018, respectively. This demonstrates the high extraction efficiency of Cu^2+^ using the investigated methodology. Moreover, the HIT Schiff base concentration of 1.57 × 10^− 5^ is considered low when compared with the reported values, which lie in the range of (6.6 × 10^− 2^ − 8 × 10^− 6^) mol.L^− 1^. Generally, the comparison of the current study results with those of the reported investigations confirms the sensitivity, reproducibility, and efficiency of the current methodology for extracting and determining the Cu^2+^.

**Table 7:** Comparative data from some recent studies on preconcentration-separation of Cu^2+^.

Organic ligand	RSD (%)	LOD	Surfactant	L (mol.L^− 1^)	Ref
PTU	< 1.4	1.6	TX-114	2 × 10^− 3^	^53^
BIYPYBI	< 0.8	1.4	TX-114	6.6 × 10^− 2^	^54^
HTAR	2–15	1.34	TX-114	8 × 10^− 6^	^1^
BrPAA	---	5.9	TX-114	1 × 10^− 3^	^55^
15-Crown-5	---	100	TX-114	9 × 10^− 3^	^56^
DDTC	3.7	0.4	TX-114	0.05% (wt/v)	^57^
DHMPhB	3.1–4.9	6	TX-100	1.5 × 10^− 4^	^58^
PAR	6.06	9.8	TX-114	2 × 10^− 4^	^59^
Poly (SMIm)-Tris-Fe_3_O_4_	3.3-5	0.093	TX-114/CTAB	---	^60^
Safranin T + pyrogallol	2.5–3.9	0.6	TX-114	1.6 × 10^− 3^ pyrogallol, 2 × 10^− 3^ Safranin T	^61^
HIT	0.270	0.018	TX-114	1.57 × 10^− 5^	This work

### The plausible mechanism of the interaction between triton X-114 surfactant and HIT-Cu^2+^ complex

**Fig. 10:**
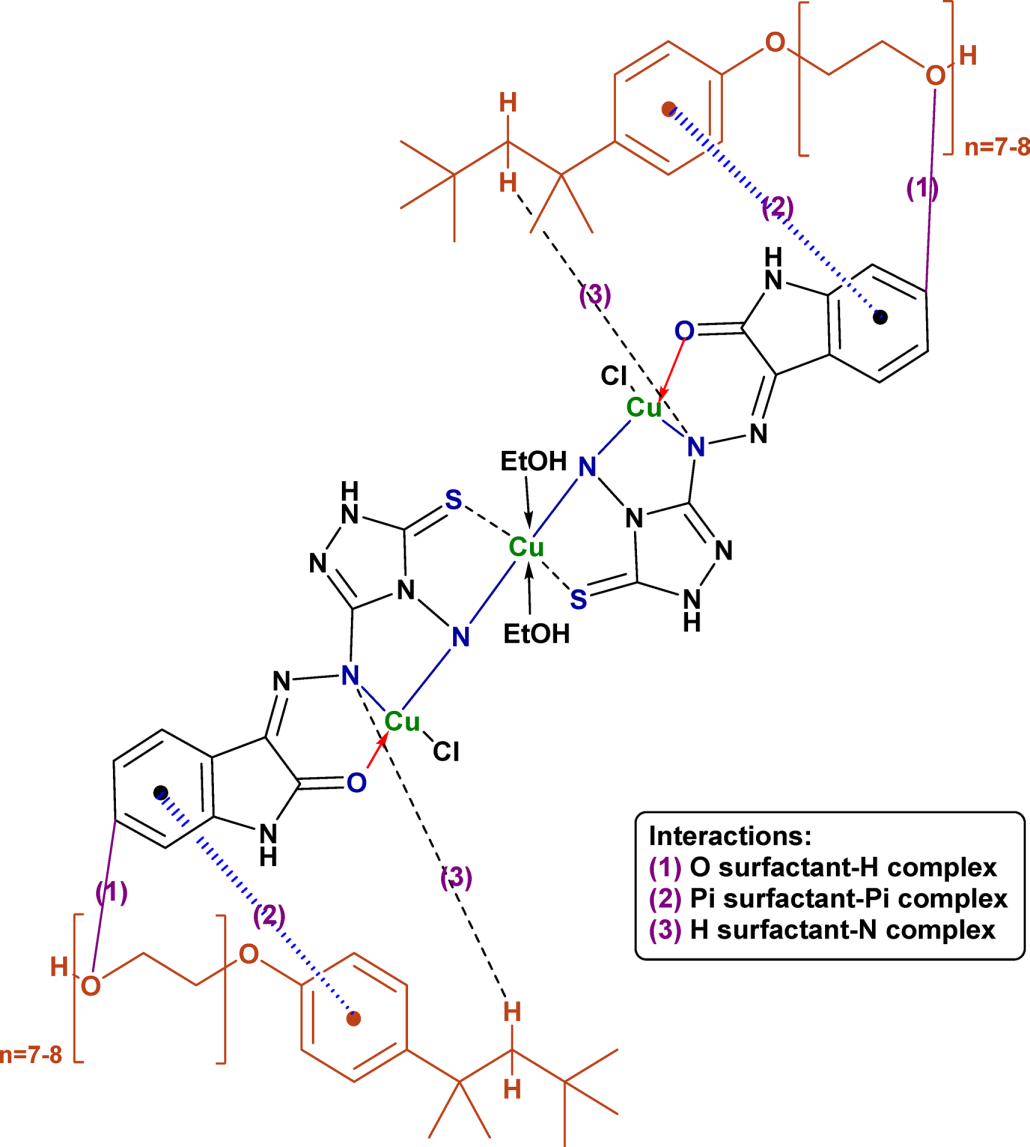
The plausible interaction between TX-114 surfactant and HIT-Cu^2+^ complex.

The plausible interaction between the HIT-Cu^2+^ complex and TX-114 surfactant is schematically illustrated in Fig. 10. As TX-114 is immiscible with H_2_O molecules, it is more favorable for effectively separating the HIT-Cu^2+^ complex from water. As presented in Fig. 10, various chemical and physical interactions occurred between the TX-114 surfactant and the HIT-Cu^2+^ complex as π-π, n-π, CH-π, hydrogen bond, and van der Waals interactions.

### Greenness evaluation

The greenness evaluation estimates environmentally friendly parameter methods, which have gained popularity in the analytical chemistry field as part of larger efforts to promote eco-friendly techniques in both research and industry. They are designed to reduce waste production and minimize the use of toxic substances and energy, while maintaining analytical performance^62^.

**Fig. 11:**
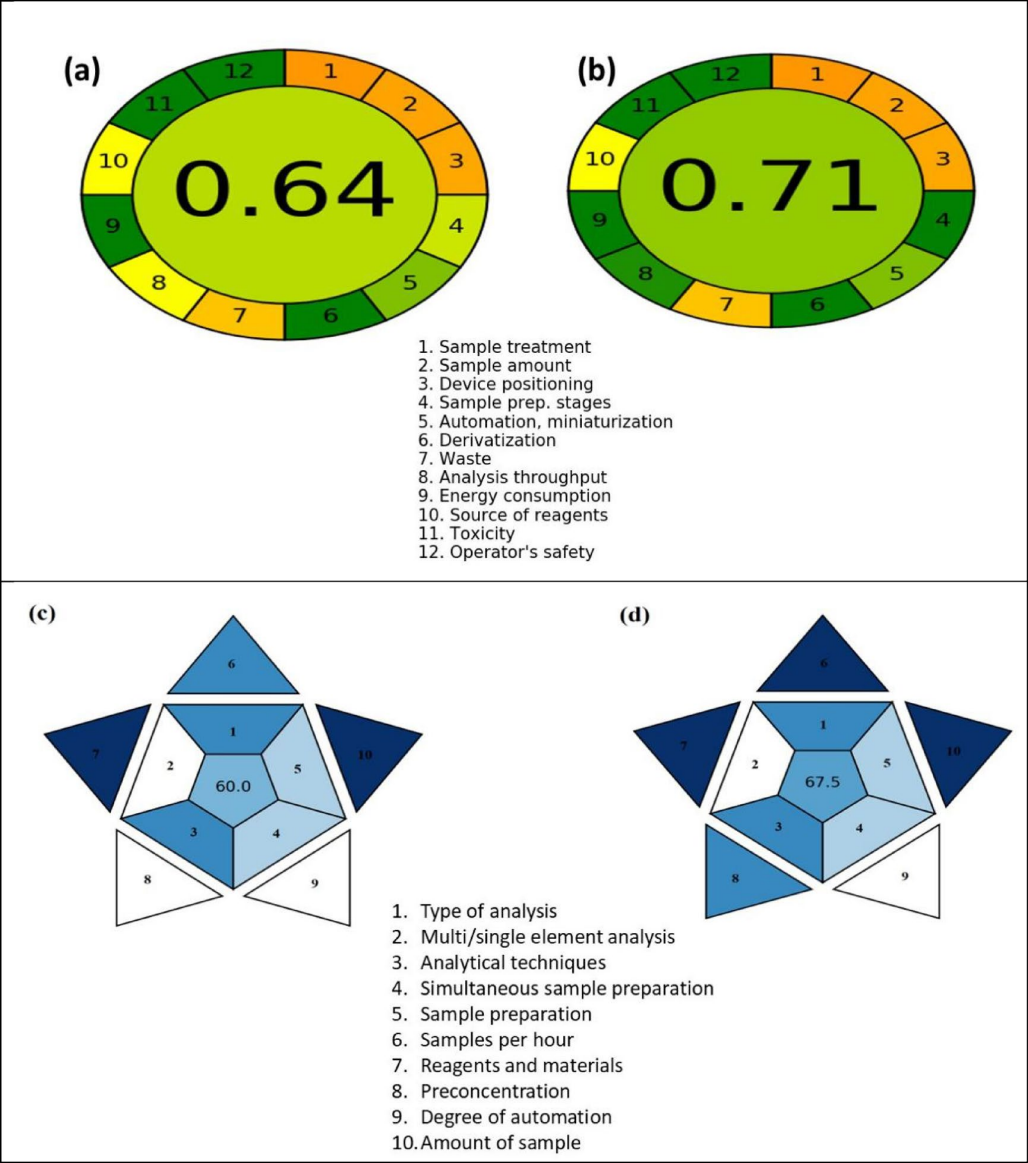
Greenness score of (**a**) CPE/ICP-OES & (**b**) RS-CPE/ICP-OES and Applicability of (**c**) CPE/ICP-OES & (**d**) RS-CPE/ICP-OES.

The greenness evaluation of the current investigated techniques, CPE/ICP-OES and RS-CPE/ICP-OES, has been investigated by the Analytical Greenness (AGREE) calculator and the Blue Applicability Green Index (BAGI). The greenness values were calculated based on two scales for comparison between the two applied methods. The AGREE tool has been utilized to assess the greenness score depending on GAC’s twelve variables that are presented in (Fig. 11a, b)^63^. The ultimate rating, which ranges from zero to one, is represented by the center of the AGREE logo. The result-related color in the center serves as the color pattern for showing the outcomes of the 12 AGREE portions. The suitable and accepted method must have a score above 0.6, indicated by a light green color^64^. The CPE/ICP-OES pictogram (Fig. 11a) had an overall score of 0.64 at its center, with no red color for any principle. Principles 6, 9, 11, and 12 showed excellent performance in green. For the RS-CPE/ICP-OES pictogram (Fig. 11b), principles 4, 6, 8, 9, 11, and 12 performed very well, with an overall score of 0.71. The AGREE scores of both CPE/ICP-OES and RS-CPE/ICP-OES, which showed no red for any principle, demonstrated their greenness and safety^64^. It was observed that the RS-CPE/ICP-OES has a higher score than that of the CPE/ICP-OES, which proves the greater greenness and safety of the RS-CPE/ICP-OES. This may be due to the decrease in time, temperature, and energy use, which resulted in this method receiving a 0.71 score for environmental efficiency. Additionally, RS-CPE/ICP-OES aids in increasing both the number of analytes in an hour and the extraction effectiveness.

The applicability of the investigated methods was determined using BAGI. The BAGI scores of 60 and 67.5 for the applied approaches demonstrated their good applicability, as shown in (Fig. 11c, d). The CPE/ICP-OES BAGI pictogram (Fig. 11c) showed three white zones corresponding to single-element analysis, low automation, and preconcentration procedures, respectively. While Fig. 11d showed the applicability enhancement with only two white zones corresponding to single-element analysis, and a low degree of automation. It was also observed that the dark green zones for RS-CPE/ICP-OES (three) were greater than those for CPE/ICP-OES (two). Moreover, both approaches showed two light blue zones for simultaneous sample preparation and the sample preparation scale^65^. As shown in Fig. 11, the score of AGREE and BAGI is higher for RS-CPE/ICP-OES (60) than that of CPE/ICP-OES (67.5). The decrease in the time consumed and the number of main steps are the main reasons for the observed enhancement. This means the application of RS-CPE not only overcame the heat and time consumption problem but also increased the greenness and applicability of the current investigation.

### Toxicity prediction

The toxicity of the HIT Schiff base and HIT-Cu^2+^ nanocomplex was predicted using PROTOX 3 software, as shown in Table 8. It was observed that the LD_50_ value for HIT and HIT-Cu^2+^ were 2100 mg.Kg^− 1^ and 1000 mg.Kg^− 1^, respectively. Which assessed that the HIT has low toxicity (class 5)^66^ while the HIT-Cu^2+^ complex has moderate toxicity (class 4)^66^. The two investigated compounds are predicted to be inactive for cytotoxicity, ecotoxicity, and immunotoxicity with high scores. While for nutritional toxicity, the HIT-Cu^2+^ complex was expected to be active with a probability of 0.57.

**Table 8:** Toxicity-related parameters for HIT schiff base and HIT-Cu^2+^ complex.

	HIT	HIT-Cu^2+^
Predicted LD_50_ (mg.kg^− 1^)	2100	1000
Predicted toxicity class	5	4
Cytotoxicity probability	Inactive 0.81	Inactive 0.79
Ecotoxicity probability	Inactive 0.53	Inactive 0.57
Immunotoxicity probability	Inactive 0.96	Inactive 0.99
Nutritional probability	Inactive 0.62	Active 0.57

### Challenges and solutions

There are many challenges facing any water treatment process. These challenges are removal efficiency, selectivity, stability, economic viability, and environmental challenges^67^. The current study has two main challenges, including time and heat (energy-consuming). It takes a long time (approximately 45 min) in addition to the optimum temperature of 50 °C. To overcome these problems, RS-CPE has been applied using decanol. The RS-CPE achieved a 100% recovery at room temperature (25 °C) without the need for heat, and the extraction time was 1 min, while the entire experiment took less than 10 min.

## Conclusion

**Fig. 12:**
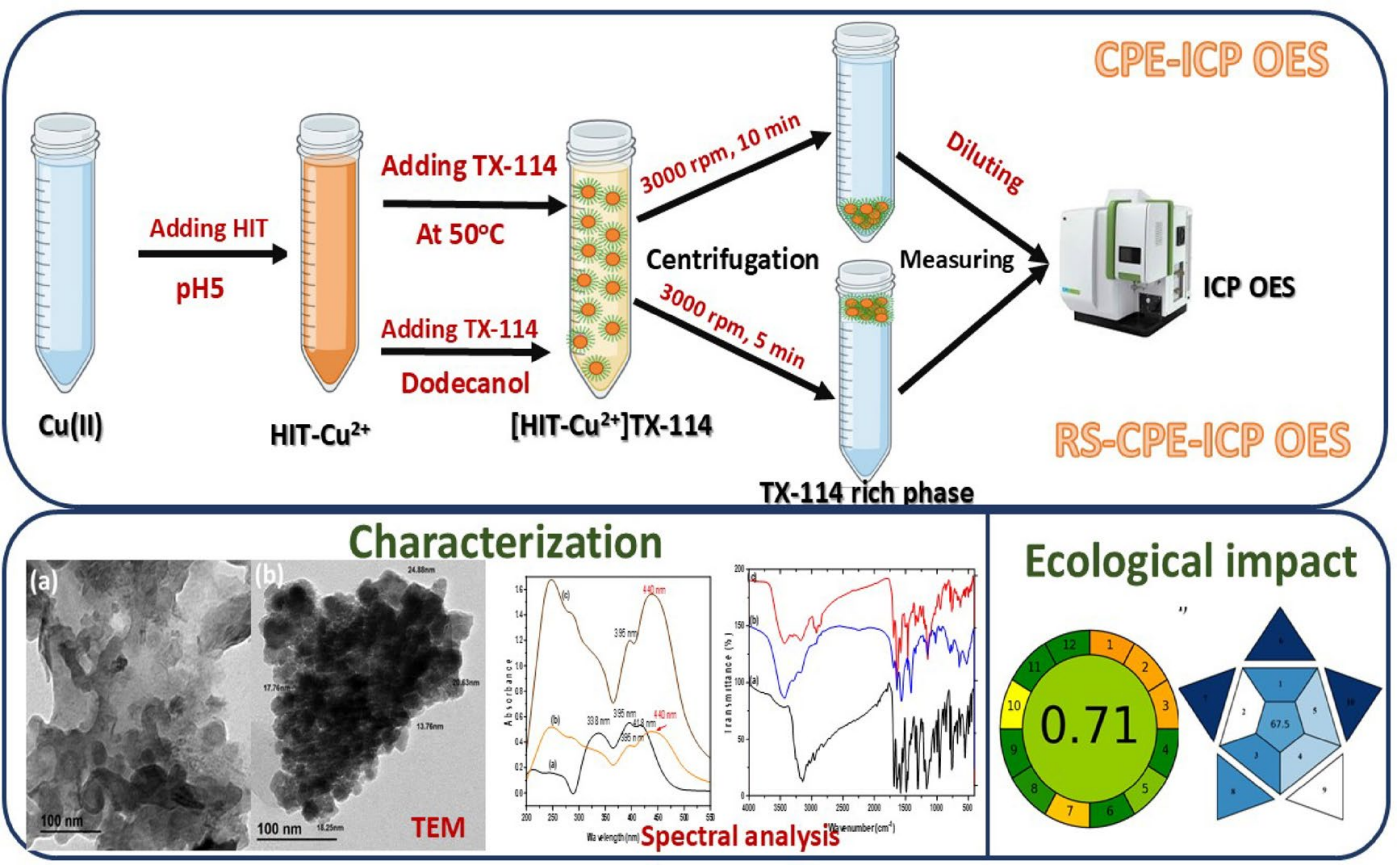
Graphical representation of the characterization of HIT Schiff base and the steps of RS-CPE/ICP-OES approach for the determination of Cu(II).

In this investigation, a comparison study was conducted between RS-CPE/ICP-OES and CPE/ICP-OES. The RS-CPE/ICP-OES performed analytically for Cu^2+^ better than the CPE/ICP-OES, as the RS-CPE/ICP-OES requires less time and does not require heating. The RS-CPE/ICP-OES showed higher AGREE and BAGI values, 0.71 and 67.5, respectively. The investigated approaches were applied for the removal, preconcentration, and determination of Cu^2+^ in various aquatic samples using the TX-114 surfactant and the HIT Schiff base. The RS-CPE/ICP-OES approach was established for examining several pharmaceutical and real water samples, and it successfully recovered more than 97% of the Cu^2+^ from spiked samples with RSD less than 1%. The toxicity of the HIT and HIT-Cu^2+^ was evaluated and found to be low and moderate, respectively. The obtained findings demonstrate the excellent accuracy, repeatability, simplicity, speed, and adaptability of the investigated technique in the Cu^2+^ preconcentration. The characterization of HIT Schiff base and the steps of RS-CPE/ICP-OES approach for the determination of Cu(II) are graphically represented in (Fig. 12).

## Supplementary Information

Below is the link to the electronic supplementary material.

Supplementary Material 1

## Data Availability

Data is provided within the manuscript or supplementary information files.
