# Supplementary material for: Rapid synergistic cloud point extraction of copper in environmental samples with greenness and toxicity evaluation using a triazole based Schiff base
Source: Sci Rep. 2026 Feb 3;16:4719. doi: 10.1038/s41598-026-35659-3 (PMC12868709; doi:10.1038/s41598-026-35659-3)
Supplement: Supplementary file 1 — Supplementary Material 1 [file 41598_2026_35659_MOESM1_ESM.docx]

**Supplementary data**

**Rapid Synergistic Cloud Point Extraction (RS-CPE) of Copper in Environmental Samples Using a Novel Triazole-Based Schiff Base: Greenness and Toxicity Evaluation**

**Magda A Akl^1^*, Eslam A. Ghaith^1^** **and Aya G Mostafa^1^**

**^1^**Department of Chemistry, Faculty of Science, Mansoura University, Mansoura 31556, Egypt

* To whom correspondence should be addressed: Prof Magda Akl.

email [magdaakl@yahoo.com](mailto:magdaakl@yahoo.com)


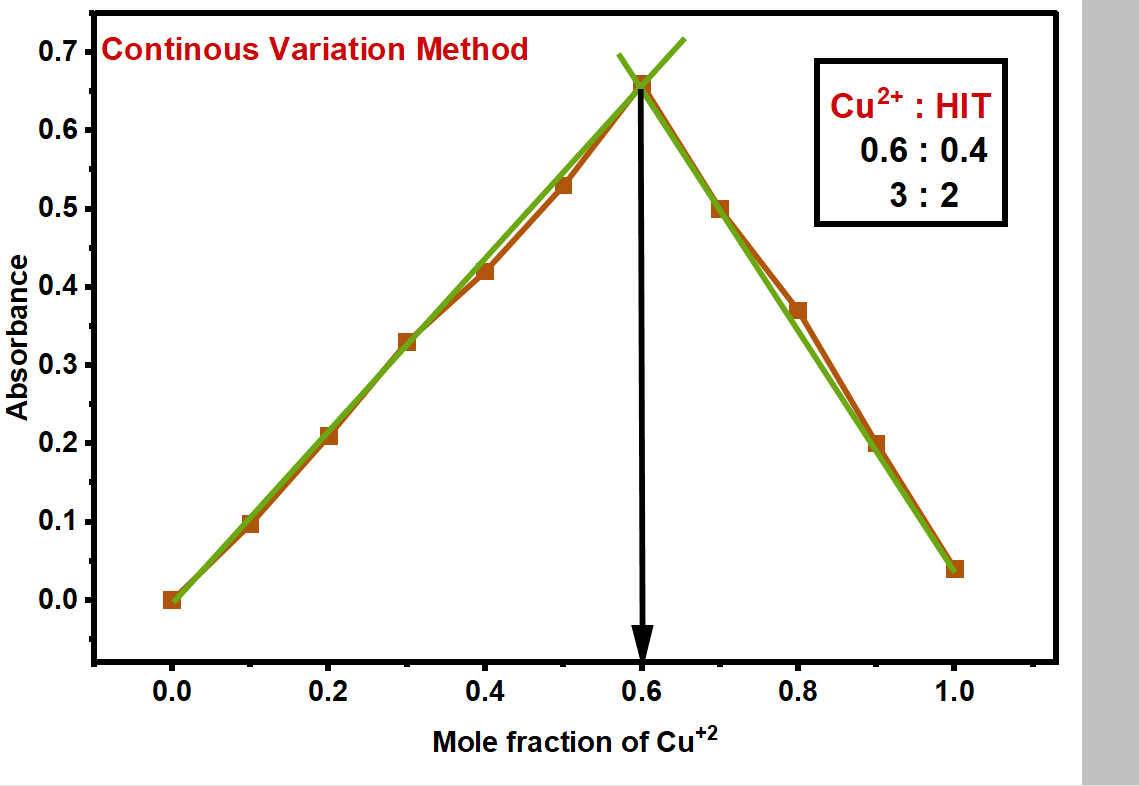


**Fig. 1S** Stoichiometric ratio
